# Glucocorticoid maintenance therapy and severe infectious complications in ANCA-associated vasculitis: a retrospective analysis

**DOI:** 10.1007/s00296-020-04752-9

**Published:** 2020-11-22

**Authors:** Claudius Speer, Christine Altenmüller-Walther, Jan Splitthoff, Christian Nusshag, Florian Kälble, Paula Reichel, Christian Morath, Martin Zeier, Raoul Bergner, Matthias Schaier

**Affiliations:** 1 Department of Nephrology, University of Heidelberg, INF 162, 69120 Heidelberg, Germany; 2 Department of Internal Medicine A, Clinical Center Ludwigshafen, Ludwigshafen, Germany

**Keywords:** ANCA-associated vasculitis, Glucocorticoids, Adverse effects

## Abstract

To study the impact of glucocorticoid maintenance dose and treatment duration on outcomes in patients with AAV (ANCA-associated vasculitis) with emphasis on infectious complications. A total of 130 AAV patients from two German vasculitis centers diagnosed between August 2004 and January 2019 treated with cyclophosphamide and glucocorticoids for induction therapy and glucocorticoids for maintenance therapy were retrospectively enrolled. We investigated the influence of glucocorticoid maintenance therapy on patient survival, time to relapse, kidney function, infectious complications and irreversible physical damage. The patients were divided into the following groups: patients treated according to the predefined reduction scheme (< 7.5 mg) or patients treated with glucocorticoids ≥ 7.5 mg after 6 months. Compared to patients receiving < 7.5 mg glucocorticoids after 6 months, patients receiving $\ge$ 7.5 mg had an increased rate of infectious episodes per patient (1.7 vs. 0.6; *p* < 0.001), including urinary tract infection (*p* = 0.007), pneumonia (*p* = 0.003), opportunistic pneumonia (*p* = 0.022) and sepsis (*p* = 0.008). Especially pneumonia during the first 24 months after disease onset [hazard ratio, 3.0 (95% CI 1.5 − 6.1)] led to more deaths from infection (*p* = 0.034). Glucocorticoid maintenance therapy after 6 months had no impact on relapse rate or patient survival and decline in kidney function was comparable. Glucocorticoid maintenance therapy with $\ge$ 7.5 mg after 6 months is associated with more severe infectious complications leading to an increased frequency of deaths from infection. Glucocorticoid maintenance therapy has no effect on time to relapse or patient survival and should therefore be critically revised throughout the aftercare of AAV patients.

**Electronic supplementary material:**

The online version of this article (10.1007/s00296-020-04752-9) contains supplementary material, which is available to authorized users.

## Introduction

The implementation of cyclophosphamide (CYC) and later rituximab (RTX) combined with glucocorticoids (GC) significantly improved outcomes of anti-neutrophil cytoplasmic autoantibody (ANCA)-associated vasculitis (AAV) during the last decades [1–3]. However, an increased immunosuppressive burden is associated with serious adverse events, including high rates of infection and malignancy with increased mortality [4, 5]. Mortality is especially increased during the first year after disease onset due to active vasculitis and infections, emphasizing the need for an accurate balance between rapid control of life-threatening disease manifestations without exposing the patients to undue risk of over-immunosuppression [6, 7]. The optimal dosing strategy for the use of maintenance GC still remains elusive. A limited GC exposure and early GC tapering were assumed to be associated with a comparable treatment response and relapse rate, but less treatment-specific adverse events, especially infectious complications [8–10]. A meta-analysis by Walsh et al. revealed a protective effect of low-dose GC maintenance therapy on the occurrence of relapses [11]. Maintenance therapies including azathioprine (AZA) or mycophenolic acid (MMF) are consequently applied to reduce the cumulative GC dose. AZA seems to be superior in maintaining AAV disease remission with adverse event rates comparable to MMF [12]. Recently, a C5a receptor inhibitor (avacopan) has been proven effective in replacing high-dose GC together with CYC or RTX in patients with milder AAV disease in a phase 2 clinical trial [13].

The aim of our study was to determine the impact of oral GC maintenance therapy with different doses and therapy duration on severe infectious complications as well as on relapse rate, patient survival, long-term kidney function and irreversible physical damage measured by Vasculitis Damage Index (VDI). We further investigated the emergence and the pathogen spectrum of infectious complications.

## Materials and methods

### Study design and population

A total of 130 patients from two different German vasculitis centers (University of Heidelberg, Department of Nephrology and the Clinical Center Ludwigshafen, Department of Internal Medicine A) with newly diagnosed granulomatosis with polyangiitis (GPA) or microscopic polyangiitis (MPA) between August 2004 and January 2019 were retrospectively included in this study: 105 patients were included at the University of Heidelberg and 25 patients at the Clinical Center Ludwigshafen. Diagnosis of GPA and MPA was made according to criteria adapted from the 1994 Chapel Hill disease definitions and based on a clinical presentation compatible with AAV together with either a positive ANCA serology and/or histology [14, 15]. The trial (ref: S-624/2014) was approved by the local ethics committee of the University of Heidelberg and conducted according to the 1964 Declaration of Helsinki and subsequent amendments. No additional approval was required for the Clinical Center Ludwigshafen according to the Landeskrankenhausgesetz (§36 and §37) of Rheinland-Pfalz, Germany.

The assessment of disease activity was performed at initial diagnosis, after 3, 6 and 12 months and during relapse. For this purpose, BVAS version 3, a score including 56 manifestations of systemic vasculitis within nine organ systems was employed [16]. Response to treatment was defined as an improvement in vasculitis manifestations indicated by a $\ge$ 50% decrease of the BVAS disease activity score. The absence of disease activity with a BVAS score of 0 and an ongoing stable maintenance immunosuppressive therapy for at least 1 month was defined as remission. Relapses were defined as new or worsened manifestations of systemic vasculitis accompanied with a BVAS score of $\ge$ 1. Refractory disease was defined as an unchanged or increased disease activity after 3 months of therapy or chronic, persistent disease with presence of at least one major or three minor items on the BVAS disease activity score despite optimized immunosuppressive treatment and dosage [17].

Patients with newly diagnosed GPA or MPA receiving IV CYC and GC for induction therapy, followed by maintenance therapy with AZA (2 mg/kg BW, orally) or MMF (2 g, orally) together with GC were included in the study (Table 1). Exclusion criteria included divergent induction and/or maintenance therapy, eosinophilic GPA, co-existent multisystem autoimmune disease and concurrent malignancy. Observation time was at least 12 months and patients with shorter follow-up were excluded from the study except for patients who died within this time period.

**Table 1:** Baseline demographics and clinical characteristics

	GC < 7.5 mg, 6 mo (*N* = 76)	GC $\ge$ 7.5 mg, 6 mo (*N* = 54)	*P*
**Diagnosis,* N*** **(%)**			
Granulomatosis with polyangiitis	42 (55)	28 (52)	0.701
Microscopic polyangiitis	34 (45)	26 (48)	0.701
Female sex,* N* (%)	38 (50)	26 (48)	0.835
BMI	25.9 (18.3–44.1)	26.0 (19.1–43.3)	0.988
Age at diagnosis, median (range), years	66 (19–82)	65 (45–84)	0.352
**Comorbidities at disease onset,* N* (%)**			
Hypertension	43 (57)	32 (59)	0.742
Diabetes type 2	15 (20)	9 (17)	0.401
Myocardial infarction	5 (7)	3 (5)	0.669
Heart failure	4 (5)	3 (5)	0.876
Chronic kidney disease (CKD $\ge$ 3a)	14 (18)	8 (15)	0.392
Active malignoma	1 (1)	0 (0)	0.901
**ANCA ELISA or IIF, *N* (%)**			
PR3	39 (51)	27 (50)	0.882
MPO	32 (42)	25 (46)	0.771
Double positive	5 (7)	2 (4)	0.474
**Organ involvement, *N*** **(%)**			
General symptoms	57 (75)	43 (80)	0.537
Ears, nose, throat	19 (25)	14 (26)	0.905
Kidney	76 (100)	54 (100)	0.999
Lung	39 (51)	31 (57)	0.492
Nerve system	6 (8)	9 (17)	0.123
Organ systems involved, median (range)	2 (1–5)	3 (1–5)	0.228
Follow-up time, median (range), mo	51 (3–116)	73 (4–144)	0.127
BVAS at disease onset, median (range)	16 (3–62)	19 (8–33)	0.106
**Kidney function at disease onset**			
Serum creatinine, median (range), mg/dl	2.4 (0.7–10.6)	3.0 (0.7–11.3)	0.122
eGFR, median (range), ml/min/1.73 m^2^	26 (4–83)	18 (7–92)	0.124
Proteinuria, *N* (%)	70 (92)	51 (94)	0.605
Hematuria, *N* (%)	69 (91)	52 (96)	0.223
Dialysis at disease onset, *N* (%)	10 (13)	9 (17)	0.577
**Induction therapy**			
Cumulative CYC dose, median (range), g	3.0 (0.8–20.8)	3.0 (0.5–6.0)	0.183
Cumulative CYC dose/kg BW, median (range), mg	39.9 (8.2–256.0)	38.4 (7.6–84.2)	0.256
Duration of CYC induction, median (range), we	12 (2–26)	12 (2–22)	0.393
Steroid pulse therapy, *N* (%)	60 (79)	41 (76)	0.683
Plasma exchange, *N* (%)	13 (17)	8 (15)	0.727
**Maintenance therapy**			
Azathioprine, *N* (%)	57 (75)	34 (63)	0.140
Mycophenolic acid, *N* (%)	8 (11)	8 (15)	0.463
Exclusively steroids, *N* (%)	11 (14)	11 (20)	0.377
Steroid dose at disease onset, median (range), mg	60 (16–80)	60 (24–80)	0.484
Steroid dose after 3 months, median (range), mg	12 (4–60)	20 (12–60)	< 0.001
Steroid dose after 6 months, median (range), mg	4 (0–8)	10 (10–40)	< 0.001
Duration of maintenance therapy, median (range), mo	35.5 (8–89)	33.0 (9–86)	0.929

*BW* body weight, *CKD* chronic kidney disease, *CYC* cyclophosphamide, *GC* glucocorticoids, *eGFR* estimated glomerular filtration rate, *IIF* indirect immunofluorescence, *mo* months, *we* weeks

Baseline data included age, gender, BMI, comorbidities at disease onset, observation time, affected organ systems and disease activity (Table 1). ANCA type was determined by ELISA or immunofluorescence. MPO-ANCA was detected by MPO-ANCA ELISA or a pANCA pattern on immunofluorescence microscopy and PR3-ANCA by PR3-ANCA ELISA or a cANCA pattern by indirect immunofluorescence microscopy. The kidney function was quantified at initial diagnosis, after 3, 6 and 12 months and after 2, 3 and 4 years by measurement of serum creatinine and glomerular filtration rate (estimated GFR, eGFR) estimated by the Modification of Diet in Renal Disease (MDRD) formula. The MDRD-GFR slope and the serum creatinine slope were defined as changes during the first 4 years compared to baseline values after obtaining stable remission. The dose as well as the total treatment time with IV CYC and GC during induction therapy was recorded. In addition, the duration of maintenance therapy with AZA or MMF together with GC was determined.

In this retrospective study, the primary objectives were patient survival, relapse-free survival and the incidence of infectious complications. Secondary objectives were kidney function, ESKD and irreversible physical damage estimated by the Vasculitis Damage Index (VDI). The VDI score is a validated checklist for irreversible physical damage which records either damage of specific organ systems affected by AAV or side effects of treatment, present for $\ge$ 3 months after disease onset [18]. We further assessed the incidence and frequency of total infectious adverse events, urinary tract infection, pneumonia, herpes infection and sepsis. The pathogen spectrum of pneumonia including opportunistic pathogens was examined separately.

To investigate the impact of the duration and dose of GC maintenance therapy on outcomes in AAV patients, we separately examined either patients treated according to the predefined reduction scheme (< 7.5 mg) or patients treated with glucocorticoids ≥ 7.5 mg 6 months after the first start of induction therapy. The standard GC maintenance scheme of both vasculitis centers (Heidelberg University Hospital and Clinical Center Ludwigshafen) is adapted to established tapering protocols [13]. The treatment regime including the induction therapy with cyclophosphamide and the GC tapering protocol are described in detail in the supplement.

### Statistical methods

Data are expressed as mean or median and range or number (*N*) and percent (%). Analysis of continuous data was performed using the nonparametric *t* test with Well’s correction or the Mann–Whitney *U* test. Statistical analysis of categorical data was performed using Chi-square test. Kaplan–Meier estimates and the log-rank test were used to determine the univariate probability of relapse-free survival, patient survival and the incidence of pneumonia during the first 24 months of follow-up. Proportional hazards models were used for relapse-free survival and incidence of pneumonia, with hazards ratios (HRs) and 95% CIs presented. Multiple logistic regression was used to detect independent associations between GC doses and the incidence of pneumonia by controlling for confounders via multivariate modeling. Statistical significance was assumed at a *P* value < 0.05. The statistical analyses were performed using GraphPad Prism version 8.4.2 (GraphPad Software, San Diego CA, USA) and SPSS version 25 (IBM, Armonk NY, USA).

## Results

### Study population and disease characteristics

Fifty-four patients were in the $\ge$ 7.5 mg GC group and 76 patients were in the < 7.5 mg GC group. At disease onset and after 3 and 6 months, patients in the < 7.5 mg group had a median GC maintenance dose of 60 mg, 12 mg and 4 mg, whereas patients in the $\ge$ 7.5 mg group had a median dose of 60 mg (*p* = 0.48), 20 mg (*p* < 0.001) and 10 mg (*p* < 0.001), respectively. Only 58% of patients achieved the dose of < 7.5 mg recommended in the GC reduction scheme. There were no differences regarding the induction therapy or the maintenance therapy (AZA versus MMF) between both groups (Table 1). The demographic and disease characteristics as well as the follow-up time were comparable although the disease activity at disease onset tended to be higher in the $\ge$ 7.5 mg group in comparison to patients receiving < 7.5 mg GC after 6 months (BVAS: 19 versus 16, *p* = 0.11) (Table 1). Comorbidities at disease onset were comparable between groups (Table 1).

### Impact of GC maintenance therapy on relapse rate and patient survival

**Fig. 1:**
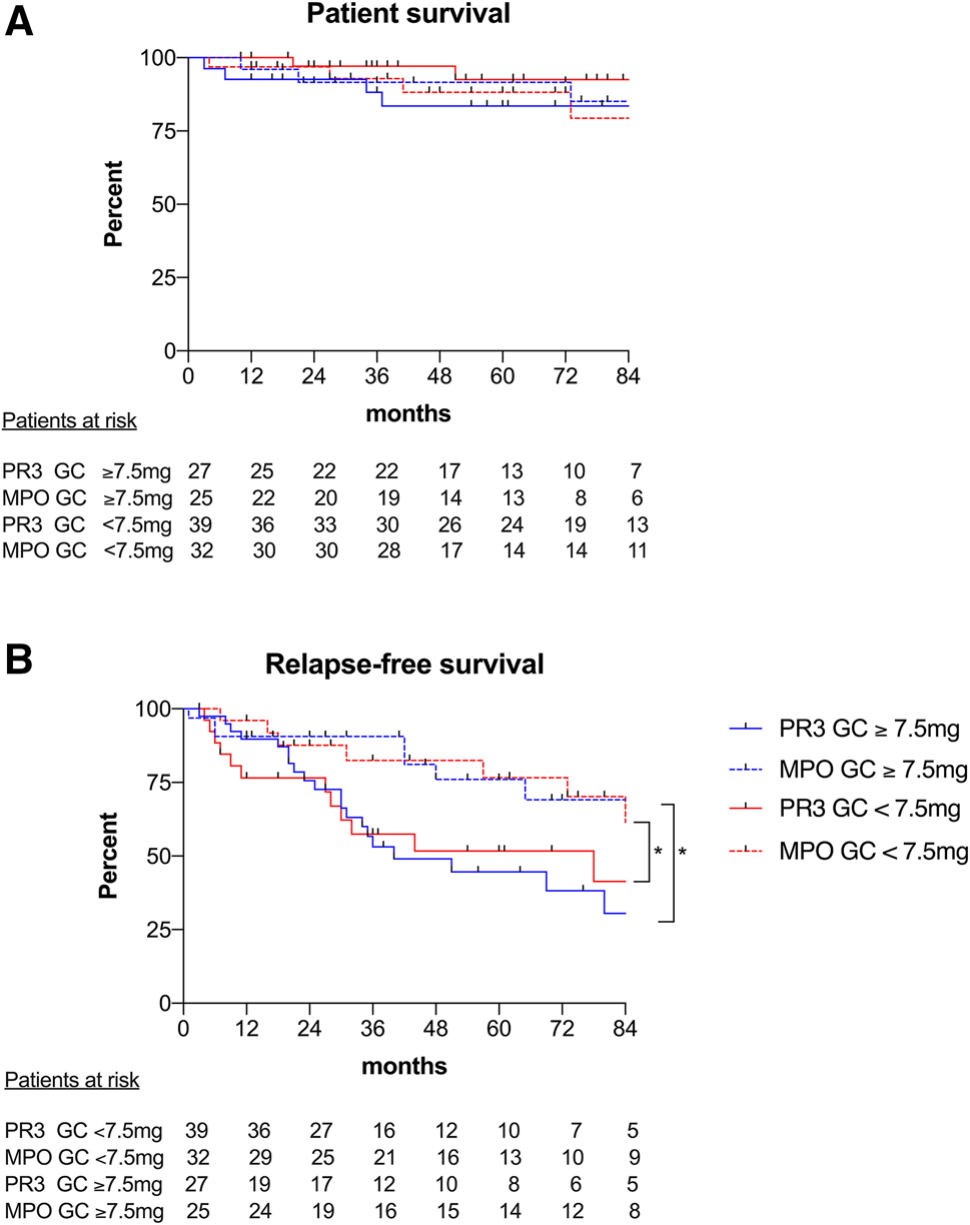
Patient survival (**a**) and relapse-free survival (**b**) in PR3-positive and MPO-positive AAV patients with a GC dose of < 7.5 mg after 6 months compared to $\ge$ 7.5 mg after 6 months

Patients with $\ge$ 7.5 mg GC maintenance therapy after 6 months showed comparable rates of patient survival (*p* = 0.31), relapse (*p* = 0.93), time to relapse (*p* = 0.80) and refractory disease (*p* = 0.67) compared to < 7.5 mg GC (Fig. 1a, b). The HR for time to relapse was 1.3 (95% CI 0.8 − 2.7) for patients with $\ge$ 7.5 mg GC compared to patients with < 7.5 mg GC maintenance therapy. GC maintenance therapy after 6 months had no impact on patient survival (Fig. 1a) or relapse-free (Fig. 1b) survival neither in PR3- nor in MPO-positive patients. PR3-positivity was associated with a significantly higher incidence of relapses compared to MPO-positivity (Fig. 1b). Three months after disease onset, disease activity tended to be higher in the $\ge$ 7.5 mg GC group (*p* = 0.09; Table 2), whereas after 6 (*p* = 0.60) and 12 months (*p* = 0.65) no difference between groups was observed. The induction therapy was comparable between the $\ge$ 7.5 mg and the < 7.5 mg GC group with a median CYC dose of 38.4 and 39.9 mg per kg BW (*p* = 0.26) and a steroid pulse therapy in 76% and 79%, respectively (*p* = 0.68; Table 1).

**Table 2:** Outcomes and complications

	GC < 7.5 mg, 6 mo (*N* = 76)	GC $\ge$ 7.5 mg, 6 mo (*N* = 54)	*P*
Relapse rate, *N* (%)	29 (38)	21 (39)	0.933
Time to relapse, median (range)	30 (1–80)	27 (5–88)	0.796
Refractory disease, *N* (%)	3 (4)	3 (6)	0.667
**Disease activity**			
BVAS after 3 mo, mean (range)	0 (0–18)	0 (0–23)	0.091
BVAS after 6 mo, mean (range)	0 (0–8)	0 (0–18)	0.600
BVAS after 12 mo, mean (range)	0 (0–17)	0 (0–3)	0.647
**Kidney function**			
Serum creatinine after 3 mo, median (range), mg/dl	1.4 (0.7–5.6)	1.6 (0.8–2.7)	0.092
eGFR after 3 mo, median (range), ml/min/1.73 m^2^	42 (9–98)	44 (19–89)	0.073
Serum creatinine after 6 mo, median (range), mg/dl	1.4 (0.7–5.3)	1.5 (0.8–2.9)	0.058
eGFR after 6 mo, median (range), ml/min/1.73 m^2^	45 (6–103)	42 (17–87)	0.073
Serum creatinine after 1 a, median (range), mg/dl	1.3 (0.8–3.7)	1.4 (0.8–3.6)	0.090
eGFR after 1 a, median (range), ml/min/1.73 m^2^	50 (8–108)	44 (22–88)	0.220
Serum creatinine after 2 a, median (range), mg/dl	1.2 (0.8–3.5)	1.4 (0.8–2.1)	0.140
eGFR after 2 a, median (range), ml/min/1.73 m^2^	53 (18–105)	48 (9–83)	0.321
Serum creatinine after 3 a, median (range), mg/dl	1.2 (0.7–3.6)	1.4 (0.7–4.1)	0.711
eGFR after 3 a, median (range), ml/min/1.73 m^2^	55 (18–130)	48 (10–92)	0.605
Serum creatinine after 4 a, median (range), mg/dl	1.1 (0.7–3.0)	1.2 (0.7–3.1)	0.531
eGFR after 4 a, median (range), ml/min/1.73 m^2^	59 (22–87)	57 (24–97)	0.568
ESKD, *N* (%)	4 (5)	6 (11)	0.220
**Infectious complications**			
At least 1 infectious complication, *N* (%)	31 (41)	41 (76)	< 0.001
Infectious episodes per patient, mean (range)	0.6 (0–7)	1.7 (0–6)	< 0.001
Urinary tract infection, *N* (%)	18 (24)	25 (46)	0.007
Pneumonia, *N* (%)	12 (16)	21 (39)	0.003
Opportunistic pneumonia, *N* (%)	2 (3)	7 (13)	0.022
Herpes virus infections, *N* (%)	7 (9)	10 (19)	0.121
Sepsis,* N* (%)	2 (3)	9 (17)	0.008
VDI after 1 year, mean (range)	0.7 (0–3)	1.4 (0–4)	0.001
Death during follow-up, *N* (%)	6 (8)	8 (15)	0.256
Death by infection, *N* (%)	1 (1)	5 (9)	0.034

*a* years, *BVAS* Birmingham Vasculitis Activity Score, *CYC* cyclophosphamide, *ESKD* end-stage kidney disease, *GC* glucocorticoids, *eGFR* estimated glomerular filtration rate, *mo* months, *VDI* Vasculitis Damage Index

### Impact of GC maintenance therapy on kidney function, infectious complications and irreversible physical damage

**Fig. 2:**
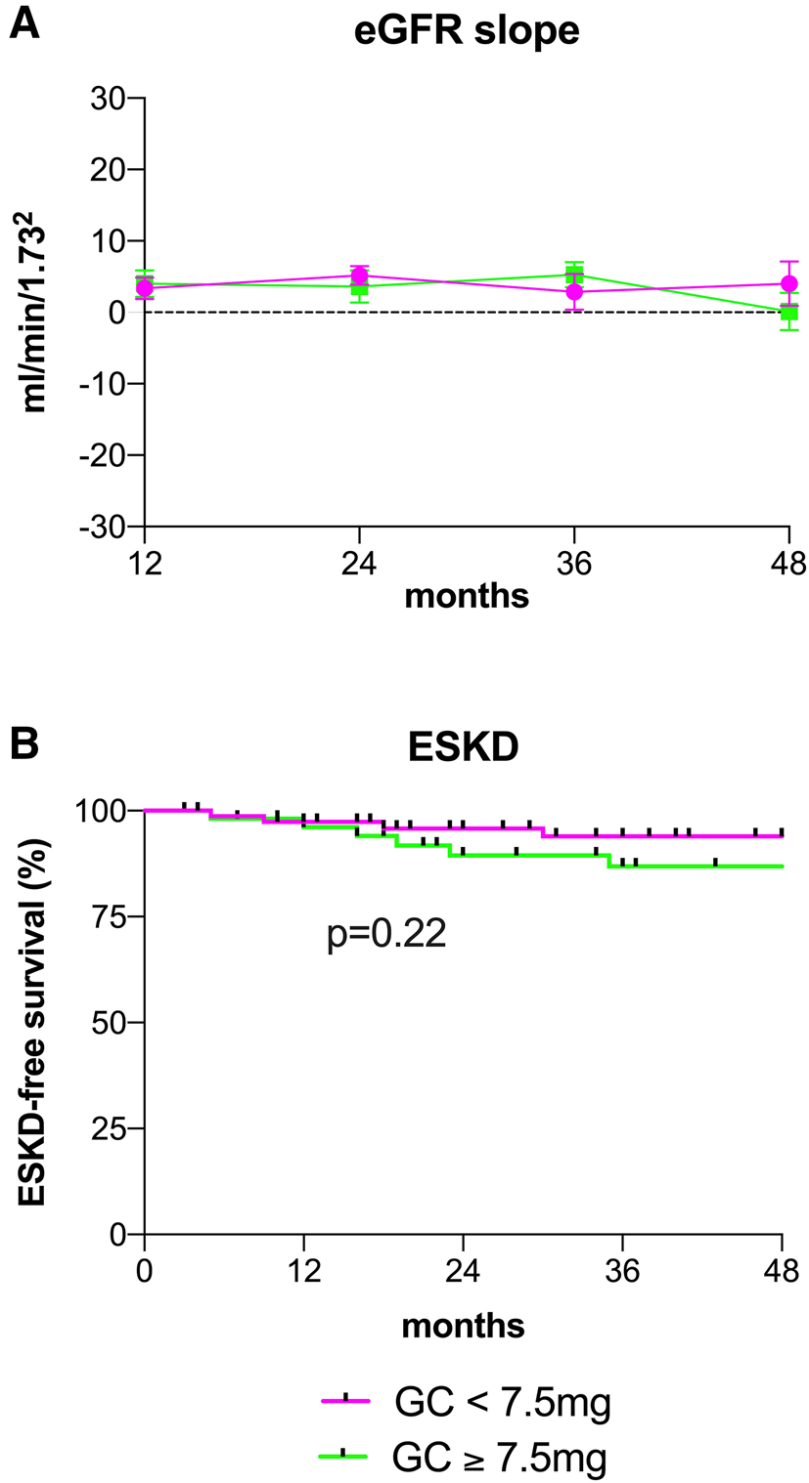
eGFR slope (**a**) and ESKD-free survival (**b**) in AAV patients with a GC doss of < 7.5 mg after 6 months compared to $\ge$ 7.5 mg after 6 months

After 4-year follow-up, kidney function as measured by eGFR (*p* = 0.57; Fig. 2a) or serum creatinine (*p* = 0.53; Table 2) and incidence of ESKD (*p* = 0.22; Fig. 2b) were not significantly different between groups.

**Fig. 3:**
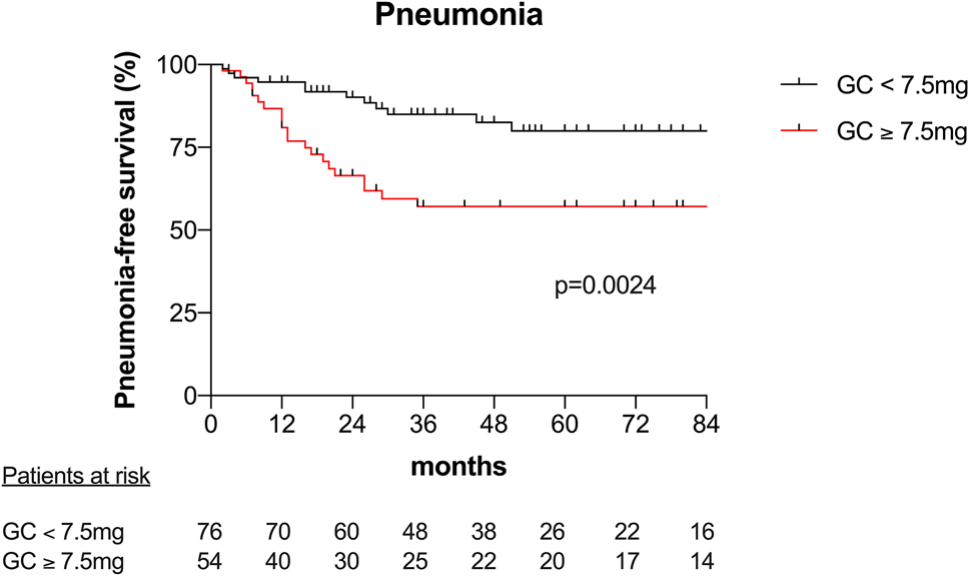
Pneumonia-free survival (%) after disease onset in AAV patients with a GC dose of < 7.5 mg after 6 months compared to $\ge$ 7.5 mg after 6 months

Infectious complications were significantly more frequent in patients with $\ge$ 7.5 mg compared to < 7.5 mg GC maintenance therapy after 6 months. At least one infectious complication occurred in 76% compared to 41% of patients (*p* < 0.001) with a median number of 1.7 versus 0.6 infectious episodes per patient (Table 2). Bacterial infectious complications such as urinary tract infection (*p* = 0.007), pneumonia (*p* = 0.003) and sepsis (*p* = 0.008) were reported more often in the $\ge$ 7.5 mg GC group, whereas herpes virus infections were not significantly different between both groups (Table 2). The incidence of pneumonia during the first 24 months after disease onset was significantly higher (log-rank *p* < 0.001) with an HR of 3.0 [95% CI 1.5 − 6.1] (Fig. 3). Because of the differences between GC groups regarding the BVAS score after 3 months (*p* = 0.09), kidney function after 6 months (eGFR; *p* = 0.07) and risk factors for developing pneumonia as lung involvement, we evaluated the impact of different GC doses on the incidence of pneumonia by controlling for these confounders by multivariate modeling. The GC dose after 6 months was the only identified factor with a statistically significant effect on incidence of pneumonia (*p* = 0.034; Table 3). Opportunistic pneumonia appeared in seven patients in the $\ge$ 7.5 mg GC group and in two patients in the < 7.5 mg GC group (*p* = 0.022; Table 2). One patient in the $\ge$ 7.5 mg group had *Pneumocystis jirovecii *(PCP), four patients *Aspergillus fumigatus*, one patient *Candida albicans* and one patient CMV pneumonia compared to one patient with *Pneumocystis jirovecii *(PCP) and one patient with *Candida glabrata* pneumonia in the < 7.5 mg group. Death by infection (*p* = 0.034) was significantly increased in the $\ge$ 7.5 mg GC group (Table 2).

**Table 3:** Logistic regression analysis of the incidence of pneumonia

Variable	OR (95% CI)	*P*
Glucocorticoid dose after 6 months	1.12 (1.02–1.21)	0.034
Lung involvement	0.49 (0.15–1.76)	0.301
BVAS score after 3 months	1.07 (0.93–1.25)	0.253
GFR after 6 months	0.97 (0.98–1.12)	0.206

*BVAS* Birmingham vasculitis activity score, *GFR* glomerular filtration rate, *OR* odds ratio, *CI* confidence interval

With a median of 1.4 versus 0.7 (*p* = 0.001), the VDI after 1 year was significantly higher in patients receiving $\ge$ 7.5 mg compared to < 7.5 mg GC after 6 months (Table 2).

## Discussion

Although the prognosis of AAV patients has improved over the last decades, mortality is still significantly higher than in to the general population due to both, active vasculitis and treatment-associated adverse events. Infectious complications are the leading cause of death during the first year after disease onset [8–10]. Efforts have been made to reduce the immunosuppressive burden either through reducing cumulative immunosuppressive dose, performing therapeutic drug monitoring or through replacing commonly used medication by more disease-specific, less toxic agents [13, 19–21]. We aimed to investigate the impact of GC maintenance dose and duration on AAV patient outcomes with an emphasis on infectious complications. This study may help to propose strategies to prevent life-threatening medication-associated adverse effects while safely controlling AAV disease activity, constituting a balancing act in the daily clinical routine.

The optimal strategy for tapering GC maintenance therapy is unknown and limiting the use of GC to the first 6 months after disease onset is not universally accepted. In a study by McGregor et al., patients were divided depending on their GC dose at 6 months into a 0 mg, 5 mg and > 5 mg GC group [9]. Among different GC groups, they found no significant differences in time to relapse. For determination of adverse events, the 5 mg and > 5 mg GC groups were combined and significantly more infectious complications were observed compared to patients without GC therapy after 6 months [9]. In the recently published PEXIVAS trial, serious infectious complications after 1 year were also less common in a reduced GC dose group than in a standard GC dose [10]. These results are in line with our study, showing comparable relapse rate but significantly increased infectious complications of patients with $\ge$ 7.5 mg GC maintenance therapy after 6 months. Except the GC therapy, maintenance medication and duration were not different between both groups. We showed that especially severe infectious complications as sepsis or pneumonia during the first 24 months after disease onset represent a significant problem for AAV patients with $\ge$ 7.5 mg GC maintenance dose after 6 months. Opportunistic pneumonia occurred more frequently, indicating that a consequent targeted prophylaxis would be advisable not only during induction therapy but also in AAV patients with higher GC maintenance dose. The VDI and death by infection were consequently higher in the $\ge$ 7.5 mg GC group. We next investigated if $\ge$ 7.5 mg GC doses after 6 months were administered due to an increased disease activity of AAV patients. Although the BVAS score tended to be higher in the $\ge$ 7.5 mg GC group after 3 months, we found no clinical differences after 6 and 12 months explaining an increased GC dose. In a recently published prospective study including 49 patients with severe AAV [22], the effectiveness of an early GC withdrawal within 7–14 days in combination with low-dose CYC and two 1 g doses of RTX was comparable to effectiveness in matched controls (*n* = 172) from previous EUVAS trials (CYCAZAREM [23], CYCLOPS [14] and MEPEX [24]). They detected no new cases of diabetes and in accordance to our study less severe infections requiring hospitalization at least after 1 year. These data are important for our cohort, since we also included severe AAV cases with pulmonal hemorrhage or dialysis at disease onset requiring plasma exchange in almost 17%. There is evidence that even in severe AAV, long-term GC maintenance therapy beyond 1 year after disease onset may not be advantageous. A systematic review and meta-analysis by Walsh et al. revealed an increased relapse rate when GC were discontinued during the first 12 months [11]. However, GC discontinuation beyond 12 months had no significant impact on the relapse rate compared to the relapse rate in patients still on GC [11]. Adverse effects of different GC regimes were not assessed in their analysis. The REMAIN study revealed an improved renal survival in AAV patients with prolonged maintenance therapy with AZA and GC to 48 months from diagnosis [25]. The prevalence and severity of adverse effects were not affected. However, GC was consequently tapered in the continuation arm with a GC dose of 5 mg per day after 12 months and 0 mg per day after 24 months [25]. Our data show that predefined steroid reduction regimens are insufficiently implemented in everyday clinical practice and that there is no reason for a higher steroid dose.

Besides attempts to reduce cumulative GC exposure, avoidance and replacement of GC by more disease-specific and less toxic agents is an upcoming and exciting research area. Recently, the C5a receptor inhibitor avacopan has been proven effective compared to high-dose GC maintenance therapy with a reduced rate of adverse events [13]. Although the treatment duration was relatively short with 12 weeks and patients with severe end-organ manifestations were excluded, avacopan may be effective in replacing GC, a finding that needs to be confirmed in a larger phase 3 study.

Our study has few limitations. First of all, the study is designed retrospectively and the patient number is relatively small. For this reason, less common side effects that are associated with GC therapy could not be assessed. In addition, the examined subgroups had different patient numbers with fewer patients in the $\ge$ 7.5 mg GC group.

This study shows that an extended glucocorticoid maintenance therapy in AAV patients induces severe infectious complications such as sepsis and pneumonia, leading to an increased frequency of death by infection. Our data conclusively indicate that GC tapering and discontinuation should be critically revised on a regular basis during the aftercare of AAV patients.

## Availability of data and material

Yes.

## Electronic supplementary material

Below is the link to the electronic supplementary material.

Supplementary File 1.

## Abbreviations

AAV: Anti-neutrophil cytoplasmic antibody-associated vasculitis
ANCA: Anti-neutrophil cytoplasmic antibody
AZA: Azathioprine
BVAS: Birmingham vasculitis activity score
BW: Body weight
CYC: Cyclophosphamide
ESKD: End-stage kidney disease
EUVAS: European Vasculitis Society
GC: Glucocorticoids
GFR: Glomerular filtration rate
GPA: Granulomatosis with polyangiitis
MMF: Mycophenolic acid
MPA: Microscopic polyangiitis
MPO: Myeloperoxidase
PCP: Pneumocystis jiroveci pneumonia
PR3: Proteinase 3
RTX: Rituximab
VDI: Vascular damage index
