# Supplementary material for: Glucocorticoid maintenance therapy and severe infectious complications in ANCA-associated vasculitis: a retrospective analysis
Source: Rheumatol Int. 2020 Nov 22;41(2):431–8. doi: 10.1007/s00296-020-04752-9 (PMC7835159; doi:10.1007/s00296-020-04752-9)
Supplement: Supplementary file 1 [file 296_2020_4752_MOESM1_ESM.docx]

**Supplemental data**

1. Cyclophosphamide induction therapy

- Cyclophosphamide 500 mg intravenously 6 times every 14 days
- Cyclophosphamide dose was adjusted according to four factors: Age, estimated GFR and white blood cell count
  - Age:
    - If <60 years old, the full dose was given
    - If 60-70 years old, the dose was reduced by 2.5 mg/kg BW
    - If >70 years old, the dose was reduced by 5 mg/kg BW
  - eGFR:
    - If >30 ml/min/1.73m^2^, the full dose was given
    - If <30 ml/min/1.73m^2^, the dose was reduced by 2.5 mg/kg BW
  - White blood cell count at the beginning of induction therapy:
    - If >4 x 10^9^/ml, the full dose was given
    - If 2-4 x 10^9^/ml, the dose was reduced by 25%
    - If <2 x 10^9^/ml, the dose was withheld until white blood cell count increased to a value above 2 x 10^9^/ml
  - White blood cell count nadir in between cyclophosphamide doses:
    - If >3 x 10^9^/ml, the full dose was given
    - If 2-3 x 10^9^/ml, the dose was reduced by 20%
    - If 1-1.9 x 10^9^/ml, the dose was reduced by 40%
    - If <1 x 10^9^/ml, the dose was withheld and further dosing was only given if white blood cell count increased to a value above >3 x 10^9^/ml

1. Glucocorticoid tapering scheme

| Study week | Glucocorticoid dose (prednisone) |
| --- | --- |
| 1 | 60 mg |
| 2 | 45 mg |
| 3 | 30 mg |
| 4-6 | 25 mg |
| 7-8 | 20 mg |
| 9-10 | 15 mg |
| 11-20 | 10 mg |
| $\geq$21 | $\leq$7.5 mg |
